# Gender Differences in Disability Life Expectancy Among Older Adults in Colombia Age 60 and Older

**DOI:** 10.1093/geroni/igaa057.1636

**Published:** 2020-12-16

**Authors:** Margarita Maria Osuna, Matthew Farina, Jennifer Ailshire

**Affiliations:** 1 University of Southern California, Los Angeles, California, United States; 2 University of Texas at Austin, Austin, Texas, United States

## Abstract

Life expectancy has increased rapidly in Colombia but it is unclear whether these added years of life are healthy years. The objective of this paper is to estimate gender differences in disability life expectancy for older adults. We use data from the first nationally representative survey of adults ages 60 and over in Colombia, the 2015 Survey of Aging, Health and Wellbeing (SABE) (N=23,694) to determine age- and gender-specific prevalence. Mortality data come from the Latin American Mortality Database. We created prevalence base life tables using the Sullivan′s method to calculate disability life expectancy. We define disability as having any reported difficulty with any of the following activities of daily living (ADL): eating, dressing, bathing, toileting, getting out of bed, and walking across a room. The age-adjusted prevalence of ADL disability was 8.7% for men and 12.4% for women. Life expectancy at age 60 for women was 20.6 years, of which 3.0 years (14%) were lived with disability. In contrast, men’s life expectancy at age 60 was 18.2 years, of which 1.7 years (10%) were lived with disability. Women have lower mortality rates than men at older ages, but because they have higher disability prevalence, they live proportionately more years with disability relative to men. We also investigated patterns at different ages, and functional limitations, and found similar gender differences. This study provides new insights into how mortality and disability patterns at older ages contribute to the gender health disparity in older Colombians.

